# Policy makers believe money motivates more than it does

**DOI:** 10.1038/s41598-024-51590-x

**Published:** 2024-01-22

**Authors:** Sebastian Jilke, Florian Keppeler, John Ternovski, Dominik Vogel, Erez Yoeli

**Affiliations:** 1 Georgetown University, 3700 O St NW, Washington, DC USA; 2 Aarhus University, Bartholins Allé 7, 8000 Aarhus C, Denmark; 3 Zeppelin University, Fallenbrunnen 3, 88045 Friedrichshafen, Germany; 4 U.S. Airforce Academy, 2354 Fairchild Drive, Air Force Academy, CO 80840 Germany; 5 University of Hamburg, Von-Melle-Park 9, 20146 Hamburg, Germany; 6 Massachusetts Institute of Technology, 100 Main Street, Cambridge, MA 02142 USA

**Keywords:** Human behaviour, Psychology and behaviour

## Abstract

To motivate contributions to public goods, should policy makers employ financial incentives like taxes, fines, subsidies, and rewards? While these are widely considered as the classic policy approach, a substantial academic literature suggests the impact of financial incentives is not always positive; they can sometimes fail or even backfire. To test whether policy makers are overly bullish about financial incentives, we asked county heads, mayors, and municipal government representatives of medium-to-large towns in Germany to predict the effects of a financial incentive on COVID-19 vaccination, and tested the exact same incentive in a field experiment involving all 41,548 inhabitants (clustered in 10,032 addresses) of the German town of Ravensburg. Whereas policy makers overwhelmingly predict that the financial incentive will increase vaccination—by 15.3 percentage points on average—the same financial incentive yielded a precisely estimated null effect on vaccination. We discuss when financial incentives are most likely to fail, and conclude that it is critical to educate policy makers on the potential pitfalls of employing financial incentives to promote contributions to public goods.

## Introduction

Motivating individuals to contribute to public goods is a central focus of public policy at every level of governance, with examples ranging from road safety^1–3^, maintenance of public spaces and institutions^4^, security of persons and property^5^, civic engagement^6^, tax compliance^7^, air and water quality^8–10^, and climate change mitigation^11^. The traditional economic approach to motivating individual contributions emphasizes the use of financial incentives^12–14^ such as prizes, fines, taxes, and subsidies. However, a growing body of scientific literature shows that when it comes to motivating contributions to public goods, financial incentives have a mixed track record. A theoretical literature suggests financial incentives can sometimes reduce people’s motivation to contribute to public goods by, e.g., interfering with non-monetary motivations such as reputation^15^. Meanwhile, an empirical literature documents a great deal of variation in their impact, and includes a perhaps surprisingly large number of examples in which financial incentives failed to promote contributions to public goods^16–24^ (e.g., when 2, 10, and 25 USD incentives failed to increase voter turnout in a 2010 election in Lancaster, CA^25^ or pay-for-performance failed to improve the work performance of U.K. public sector employees^26^), as well as some striking examples in which financial incentives backfired^18,27^ (e.g., when the introduction of performance pay led volunteers to work less hard^28^, or the introduction of a fine led to less compliance^29^).

Little is known about policy makers’ knowledge about the effectiveness of such financial incentives. A systematic review reveals no research on this topic (see SM, Section 2.1). There are reasons to believe that policy makers are not accounting for the potential pitfalls identified in the literature. One reason is that neoclassical approaches remain central in policy education. For instance, we reviewed curricula of all 17 public management programs for federal and state civil servants and the top 10 law schools in Germany—two of the most popular degrees held by German mayors^30,31^—and found that none of these curricula included the above ‘behavioral’ literature on the potential limitations of financial incentives. Another reason is that policy makers make frequent and ongoing use of financial incentives to promote contributions to public goods. Examples include the prominent role of material rewards in the Chinese social credit system^32^, subsidies for the adoption of green technologies within the U.S.’s 2022 infrastructure bill^33^, Germany’s suite of energy efficiency and electric vehicle subsidies^34^, as well as vaccine lotteries in many areas of the United States^35^. Finally, there are a number of behavioral and organizational barriers and biases that might slow adoption of evidence related to financial incentives, e.g., it is difficult to evaluate such evidence^36^, it is common to place too much weight on positive effects or local experts’ perspectives^37,38^, it is more natural to rely more upon prior attitudes and beliefs than on research^39^, political beliefs can influence how one views the research^40^, or, even upon updating one’s own beliefs, it might be hard to adopt evidence due to organizational inertia^41^. The lack of policy education about the potential pitfalls of financial incentives, incentives’ prominent role in policy, and the plethora of barriers and biases standing in the way of evidence adoption all suggest that policy makers might place more confidence in the impact of financial incentives than is warranted by the scientific consensus.

In this study, we present a stark example in which one should be uncertain about whether financial incentives would successfully motivate contributions to a public good, yet policy makers expected they would work. We focus on the case of COVID-19 vaccination. This is a setting where the scientific literature would suggest uncertainty about the effectiveness of financial incentives. Empirically, while there have been several high-profile studies^42,43^ that have found financial incentives to be effective in increasing COVID-19 vaccination, one meta-analysis^44^ and one systematic review^45^ have found that financial incentives’ impacts on COVID-19 vaccination varied substantially, having had a small positive effect on average across all studies, but having had no impact in a relatively large share of studies. The meta-analysis concludes that the effect sizes of financial and non-financial treatments do not differ significantly^44^. In a few cases, financial incentives even backfired^46–48^. There are also theoretical reasons to believe that financial incentives may have limited effectiveness in this context. First, it is a setting where concerns over the public good are paramount: although vaccination confers clear benefits to the individual, there are also important benefits to the public good, via reduced transmission^49–51^ and mutation of the virus^52–55^. Moreover, a goal of public policy in this domain is to motivate individuals to vaccinate even when they might not have done so purely out of concern for their own health^56^. Second, non-financial motivations in general, and reputational motivations in particular play an outsized role in motivating COVID-19 vaccination^57^ and evasion thereof^58^. For instance, in survey data from Germany, 65.8% of respondents cited *“I want to contribute to eradicating the virus”* as one of their top three motivations for vaccination, and 21.2% cited *“My social environment exerts pressure”*^59^. Third, proposed financial incentives for vaccination tend to be relatively small—typically the equivalent of about 25 USD. For instance, one prominent study employed a 20 Euro incentive in increasing vaccination^43^, and the author team themselves characterized this incentive as “small.” As has been argued^15^ and shown empirically^28^, moderate to small financial incentives can fail or backfire because they can undermine other motivations without providing sufficient motivation of their own right^15,29^. Finally, financial incentives for vaccinations may backfire because they lead people to infer that the vaccine is risky. Zhang and Lane^47^ found that even large monetary incentives reduced Chinese nationals’ willingness to get vaccinated against COVID-19 and they speculated the monetary incentives were construed as signals about the vaccine’s safety, and in a study of willingness to participate in a clinical trial, increasing financial incentives for participation increased the prospective participants’ perception of study risk^60^.

Yet, in the midst of the COVID-19 pandemic, as governments at all levels considered policies to motivate vaccination against the virus, financial incentives were prominently discussed and promoted by numerous academics^61,62^, policy makers^63^ including by prominent public health institutions such as the US Centers for Disease Control and Prevention^64^, in the media^65,66^, and even in congressional testimony^67^. In prediction studies with scientific experts, financial incentives were predicted to be particularly effective^61^. Many localities ultimately implemented financial incentives^68^, including nearly half of U.S. states^68^ and major cities such as Philadelphia^69^; Ohio and California are noteworthy in that they offered large lottery incentives of up to 1–1.5 million USD, respectively^68^.

During this period, our team collaborated with the German town of Ravensburg to test policy makers’ predictions of a policy that relied on financial incentives to promote COVID-19 vaccination. We also evaluated the actual impact of this policy on vaccination uptake via a randomized controlled trial using the entire population of Ravensburg. Ravensburg is a city in southern Germany, in the state of Baden-Wuerttemberg, approximately two hours by car or train from Munich, Stuttgart, and Zurich. It has 41,548 adult inhabitants living in 10,032 distinct addresses. The mean net household income is 49,107 Euros, 2.7 percent above Germany’s national average^70^. In November and December 2021, Ravensburg organized a series of seven COVID-19 vaccination events and an accompanying marketing campaign including a personalized letter to every adult resident to advertise the events. At the time the city commenced its campaign, most residents were eligible to be vaccinated or boosted: the vaccination rate in Ravensburg and the surrounding county was 61% and the administration of booster vaccinations just started for people 70 years and older, and high-risk groups (these rates were below those of the state and Germany as a whole, which were 62.8%^71^ and 66.8%^72^, respectively). In the hopes of motivating first and follow-up (booster) vaccination, Ravensburg recruited corporate donations, which it used to provide incentives of 20 Euros (approximately $20 USD) per vaccination—88% of Germany’s median hourly wage, and approximately double the minimum wage at the time^73^. We also included a community-level financial incentive in the letters, which we discuss in detail in the Supplemental Materials (SM, Section 1.2). This incentive meant that, if enough people attended the vaccination events, the financial incentive would be doubled to 40 Euros; this threshold was ultimately met and people were indeed paid 40 Euros for vaccinating. The city’s total cost for these financial incentives amounted to 6,640 Euros, roughly 9% of the total budget for the vaccination drive (the city incurred costs of approximately 42,000 Euros in printing and mailing costs, and 30,000 Euros to staff and secure the events). The vaccination events were advertised via letters, designed according to best practices^74^, signed by the mayor and two local health officials, and mailed to each resident. There were two versions of the letters: one which included a voucher which entitled the recipient to the financial incentive, and one which did not.

**Figure 1:**
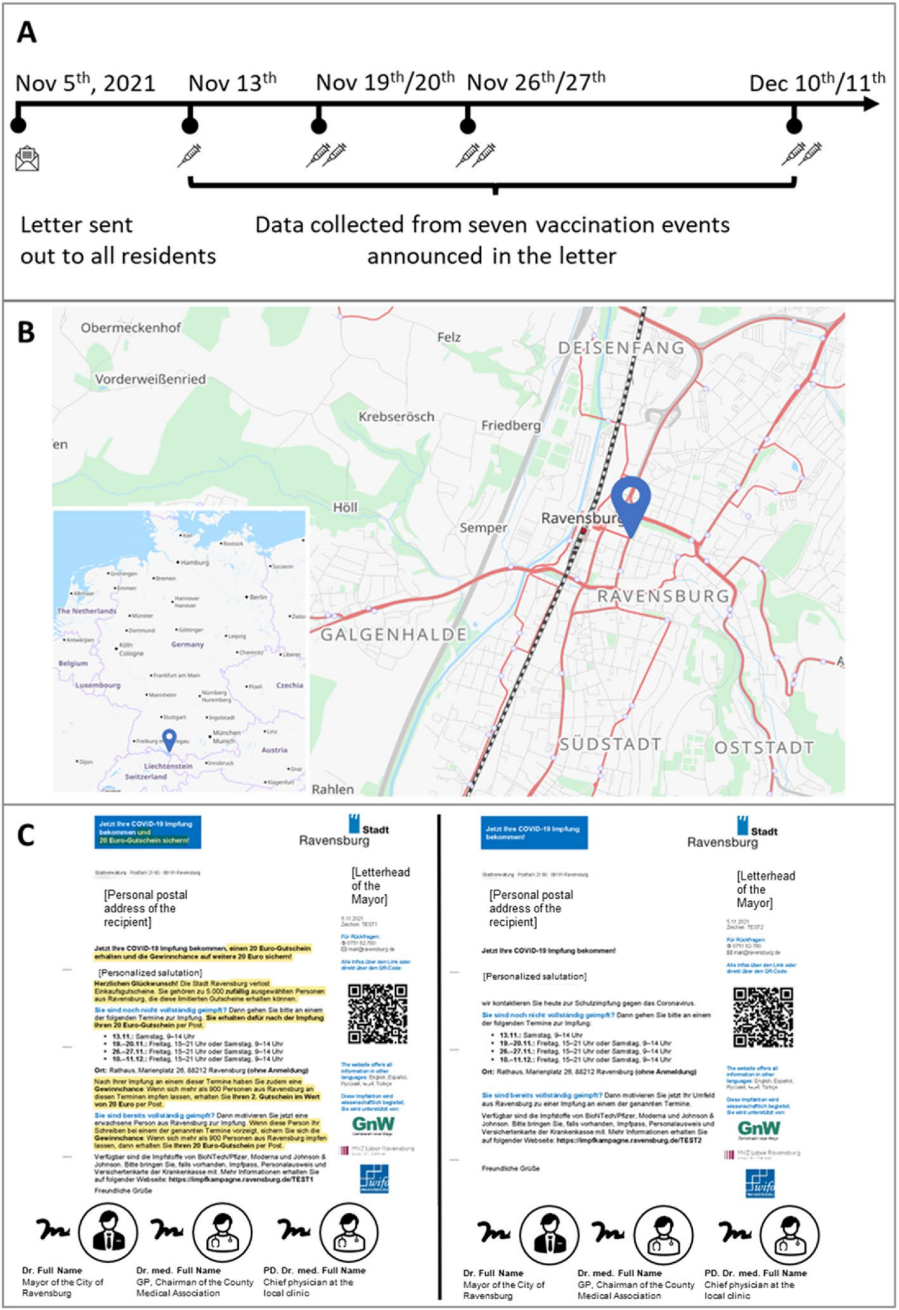
Randomized Control Trial of the Effect of a Financial Incentive on COVID-19 Vaccination. We sent all 41,548 adult residents of the town of Ravensburg, Germany an invitation to be vaccinated at one of seven vaccination events, randomly assigning 5,016 individuals to receive a 20 Euro financial incentive. (**A**) Timeline of the invitations and events. (**B**) Map of Ravensburg indicating the location of the vaccination events. (**C**) Reproductions of the invitation letters. Left: Letter sent to the intervention group, which offered the 20 Euro financial incentive. The text associated with the incentive is highlighted in yellow. Right: Letter sent to the control group, which is identical except that it omits text associated with the financial incentive. For a translation of these letters into English, see SM, Sect. 1.3.

In addition to the incentive-based randomized evaluation, and consistent with recommendations in the literature^75^, we conducted a prediction exercise in which we asked experts to predict whether the financial incentive would successfully motivate vaccination in Ravensburg. Crucially, we targeted local policy makers with the authority to implement interventions in their localities, contacting 815 county heads and mayors or municipal government representatives of towns with 30,000 or more residents in Germany—these are policy experts who have the authority and capacity to implement policies similar to the Ravensburg vaccination campaign. In our prediction exercise, we explained that Ravensburg was testing the effect of financial incentives on vaccination in a randomized control trial (RCT), and stated the exact dates when the RCT was implemented. After providing some relevant background information including the rate of first, second, and booster vaccinations, we showed participants copies of both letters (see Fig. 1), and, for each, asked respondents to use their experience as local policy makers to predict “what percentage of adult residents who received a letter *with* [*without*] a voucher got vaccinated on one of the seven dates?”. We then asked the same question, but for booster shots (for the survey instrument in German and English, see SM, Section 2.5). Survey responses were anonymous, and participants were made aware of this.

## Results

Of the policy makers contacted, 10.8% (i.e., 88 in total) completed our prediction exercise (for more detail, see SM, Section 2.4). Respondents in our prediction study reported having authority over at least 5.7 million German residents. Overwhelmingly, policy experts believed that financial incentives would meaningfully increase vaccinations in Ravensburg. On average, they estimated that 27.5% of adults who were not offered the incentive would get vaccinated, whereas 42.8% who were offered the incentive would get vaccinated, a difference of 15.3 percentage points (*SD *= 15.7, *t*(86) = − 9.06, *p *< .001; for an alternative specification of this analysis, please see SM, Section 2.3 for details). Responses for booster shots displayed a similar pattern: respondents predicted that 32.3% of adults who were not offered the incentive would get boosted, whereas 48.2% of adults who were offered the incentive would get boosted, a difference of 15.9 percentage points (*SD *= 17.7, *t*(87) = − 8.38, *p *< .001). Only twelve (13.8%) respondents expected a null effect of financial incentives, and four (4.6%) a negative effect. For boosters, 13 (14.8%) expected a null effect, and two (2.3%) a negative effect.

To test whether financial incentives actually motivated vaccination, we worked with Ravensburg to implement a preregistered RCT testing the same letters that were presented in the survey (see Fig. 1). We randomly assigned half of Ravensburg’s 10,032 unique addresses to be eligible for a 20 Euro financial incentive. Then, in these addresses, we randomly selected a single adult in the household to receive the financial incentive (N = 5016; intervention group). All other adults received an identical letter without the incentive (N = 36,532; control group). Our power calculations suggested that we would be able to detect a difference in vaccination rates of 1.3 percentage points across the two groups with 80% power. See SM, Sections 1.2, 1.4, and 1.9 for further details on the experimental design and power calculations.

**Figure 2:**
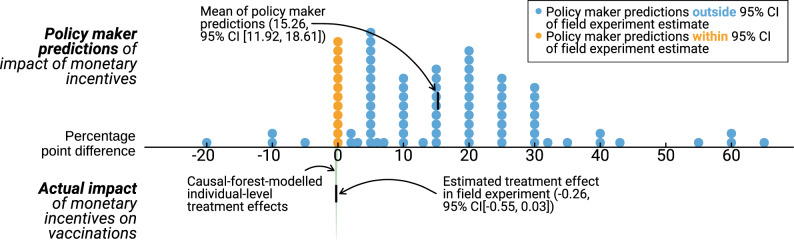
Policy Makers’ Estimates of the Effects of Financial Incentives Compared to the Actual Effects of the Incentive. We asked German mayors and other policy makers to predict “what percentage of adult residents who received a letter *with* [*without*] a voucher got vaccinated on one of the seven dates?”. Here, we present their estimates of the increase in vaccination rates from our 20 Euro financial incentive [as a percentage point difference of their estimate of the vaccination rate in the control group]. The average estimated increase was 15.3 percentage points (119.1%). Estimates highlighted in orange fall within the 95% confidence interval of the actual increase in the randomized trial (− 0.26 percentage points, 95% CI [− 0.55, 0.03]). For comparison, the green density plot presents estimated individual treatment effects for all of Ravensburg’s 41,548 adult residents from a causal forest^76^. We find no evidence of treatment effect heterogeneity in our field experiment (an omnibus significance test fails to reject the null hypothesis of homogeneity at *p* value = 0.93^76^). We include the causal forest predictions to illustrate that even with some underlying treatment effect heterogeneity, policy makers overwhelmingly fail to predict the actual treatment effects.

In stark contrast to policy makers’ predictions, we found no effect of financial incentives on vaccination (see Fig. 2). The proportion of Ravensburg residents assigned to the control group who were vaccinated at one of the seven vaccination events was 1.94%. In the intervention group, 1.73% of residents were vaccinated at the events. The difference in vaccination rates between individuals that received the intervention and individuals that did not was − 0.26 percentage points when controlling for the covariates we pre-registered (95% CI [− 0.553, 0.027], *p *= .076) (see Table S5 for alternative specifications that yield similar results). When we compare policy makers’ predictions to these results, we see that, for instance, over 86% of policy makers’ predictions were outside the 95% confidence interval of the randomized trial, and that over 95% of policy makers’ predictions were greater than the 75th percentile of estimated individual effects.

One possible explanation for the discrepancy between policy makers’ predictions and the results of the field trial is that in making their predictions, policy makers’ focus on a particular constituency that is not representative of the average. Thus, we investigated the possibility that incentives work for some residents but not others. We found no evidence that this was the case: the effect of the incentives does not significantly vary by residents’ age, gender, and nationality. We test for heterogeneity using an omnibus treatment effect heterogeneity significance test^76^; we fail to reject the null hypothesis of treatment effect homogeneity at *p* value = .93 (see SM, Section 1.5.3 for further details). We also consider, in particular, the possibility that the policy worked just for first-time vaccinations or for boosters, especially since individuals who had not yet obtained a first vaccination by this point might have been vaccine-hesitant or otherwise more difficult to motivate to obtain a vaccine. However, we found a null result for first-time vaccinations (-0.02 percentage and a small but statistically significant *negative* effect for boosters vaccinations (-0.30 percentage points, *p* value = .006; see SM, Table S2 for more detail on both).

Another possibility is that policy makers did not have a precise sense of the impacts of interventions on vaccination, because, for instance, they find it difficult to mentally simulate their effects or have poor intuitions for statistics^77–83^; our comprehensive review of the curricula of public management programs and law schools revealed little in terms of statistical training. Consistent with this, we find that policy makers did not accurately predict the ‘base’ response rate in the control group. This finding is shared with those of other, recent prediction studies^69,83–85^. In such cases, it can be informative to consider alternative outcome measures, such as relative rankings, which provide an indication of which intervention the respondent expects to work better. These are reported above. Another outcome that is often used in such cases^69,83–85^ is percentage changes—relative to the base rate—which provide some indication of whether the respondent thought the difference between the interventions would be, relatively speaking, big or small. We, therefore, repeat our main analysis using percentage changes and find similar results: most policy makers expected a big boost in the vaccination rate amongst those who received the incentive, not just in absolute terms, but also relative to their guess of the base rate (see SM, Section 1.5.2 for details).

## Discussion

Overall, these findings show policy makers drastically over-predicted the effect that financial incentives had on contributions to a public good in a high-stakes setting, earmarking 6,640 Euros, or roughly 9% of the total vaccine drive budget to incentives that ultimately proved completely ineffective. To the extent that these results are indicative of a broader tendency by policy makers to rely on financial incentives when they are likely to fail, we emphasize the importance of educating policy makers on the potential pitfalls of employing financial incentives when attempting to promote contributions to public goods. Doing so should, in our view, be a major focus of policy communication.

While our results and discussion have emphasized that financial incentives can fail to promote contributions to public goods, they do not always fail^43,86^, nor are they always expected to. The literature points to two key conditions that reduce the likelihood financial incentives will work. The first is whether the behavior involves a contribution to the public good. Particularly for contributions to public goods, one should be cautious when considering employing financial incentives. If, however, the behavior in question involves a mostly personal decision (e.g., whether to lose weight ^87^, go to the gym ^88^, sleep more ^89^, or to be screened for cancer^90^) then there are fewer concerns with using financial incentives. This is because non-financial motivations such as reputation are particularly important in motivating contributions to public goods^91–93^, and these can be ‘crowded out’ by financial incentives^27–29^. This is admittedly complicated by the fact that the same behavior may simultaneously involve private benefits while also being a contribution to the public good, as is the case for vaccination^59^; when this is the case, one should try to anticipate people’s motivations, e.g., whether most people are primarily motivated to protect themselves from COVID-19, or whether they are not so worried about COVID-19 for themselves, and might instead be motivated by the protective effects of vaccination on others, and the potential to achieve herd immunity.

Second, the literature points to *small* financial incentives as particularly likely to fail or backfire. To quote the title of one well-known paper, it is advised to “pay enough or don’t pay at all” ^28^. This is because, to be effective, incentives must be sufficiently large to not only compensate individuals for the costs—in time, effort, discomfort, *etc.*—of contributing to the public good, but also to make up for the fact that these incentives undermine other, non-financial motives for contributing. Relatively large incentives have been shown to work where smaller incentives fail. For instance, in a field experiment, paying volunteers 1% of the revenue they raised led them to work less hard than when they received no payment, but paying 10% of revenue increased their effort^28^. Many of the financial incentives for adoption of electric vehicles and energy-efficient technologies in the U.S., Europe, and Asia are quite large (e.g., Germany offers a subsidy of up to 6,000 Euros per electric vehicle). Such large subsidies are likely large enough to be effective; in the past, incentives of this magnitude have been successful at motivating uptake of hybrid and electric vehicles^94,95^, as well as energy efficiency technologies such as heat pumps^96^.

### Ethics

The Zeppelin University ethics committee reviewed and approved this study on 29th October 2022. All vaccination events were conducted in strict adherence to the vaccination procedures stipulated by the German public health authorities, which includes a mandatory consultation by a doctor prior to vaccination and the participant’s signed confirmation of consent to get vaccinated. The Zeppelin University ethics committee waived participants’ informed consent as it relates to being included in the study and the collection of data as to whether or not a participant was observed consenting to be vaccinated at the public event. The study was performed in accordance with relevant guidelines and regulations.

### Supplementary Information

Supplementary Information.

## Data Availability

An anonymized version of the data generated and analyzed for this study are available in the OSF repository, https://osf.io/uh2sd/. Links to preregistration: 10.17605/OSF.IO/2TRMB and 10.1186/ISRCTN59503725.

## References

[1] Khayesi M, Peden M (2005). Road safety in africa. BMJ.

[2] World Health Organization, Global status report on road safety: Time for action (2009). Accessed at https://www.afro.who.int/publications/global-status-report-road-safety-time-action on October 17, 2022.

[3] Belin M-A, Tillgren P, Vedung E (2012). Vision zero–a road safety policy innovation. Int. J. Injury Control Saf. Promot..

[4] Ostrom E (1990). Governing the Commons: The Evolution of Institutions for Collective Action.

[5] Bennett T, Holloway K, Farrington D (2008). The effectiveness of neighborhood watch. Campbell Syst. Rev..

[6] Green DP, Gerber AS (2019). Get out the Vote: How to Increase Voter Turnout.

[7] Torgler B (2003). To evade taxes or not to evade: That is the question. J. Socio-Econ..

[8] Greenstone M (2002). The impacts of environmental regulations on industrial activity: Evidence from the 1970 and 1977 Clean Air Act amendments and the census of manufactures. J. Polit. Econ..

[9] Melnick RS (2010). Regulation and the Courts: The Case of the Clean Air Act.

[10] Greenstone M, Hanna R (2014). Environmental regulations, air and water pollution, and infant mortality in India. Am. Econ. Rev..

[11] Nordhaus W (2019). Climate change: The ultimate challenge for economics. Am. Econ. Rev..

[12] Pigou AC (1947). A Study in Public Finance.

[13] Bovenberg, A. L. & Goulder, L. H. *Handbook of Public Economics* (Elsevier, New York, 2002), vol. 3, pp. 1471–1545.

[14] Becker GS (1968). The Economic Dimensions of Crime.

[15] Bénabou R, Tirole J (2006). Incentives and prosocial behavior. Am. Econ. Rev..

[16] Karlan D, List JA (2007). Does price matter in charitable giving? Evidence from a large-scale natural field experiment. Am. Econ. Rev..

[17] Rondeau D, List JA (2008). Matching and challenge gifts to charity: Evidence from laboratory and natural field experiments. Exp. Econ..

[18] Karlan D, List JA, Shafir E (2011). Small matches and charitable giving: Evidence from a natural field experiment. J. Public Econ..

[19] Lacetera N, Macis M, Slonim R (2012). Will there be blood? Incentives and displacement effects in pro-social behavior. Am. Econ. J.: Econ. Policy.

[20] Ashraf N, Bandiera O, Jack BK (2014). No margin, no mission? A field experiment on incentives for public service delivery. J. Public Econ..

[21] Noussair CN, van Soest D, Stoop J (2015). Punishment, reward, and cooperation in a framed field experiment. Soc. Choice Welfare.

[22] Kraft-Todd G, Yoeli E, Bhanot S, Rand D (2015). Promoting cooperation in the field. Curr. Opin. Behav. Sci..

[23] Gangl K, Torgler B (2020). How to achieve tax compliance by the wealthy: A review of the literature and agenda for policy. Soc. Issues Policy Rev..

[24] Goette L, Stutzer A (2020). Blood donations and incentives: Evidence from a field experiment. J. Econ. Behav. Organ..

[25] Panagopoulos C (2013). Extrinsic rewards, intrinsic motivation and voting. J. Polit..

[26] Burgess S, Ratto M (2003). The role of incentives in the public sector: Issues and evidence. Oxford Rev. Econ. Policy.

[27] Frey BS, Oberholzer-Gee F (1997). The cost of price incentives: An empirical analysis of motivation crowding-out. Am. Econ. Rev..

[28] Gneezy U, Rustichini A (2000). Pay enough or don’t pay at all. Q. J. Econ..

[29] Gneezy U, Rustichini A (2000). A fine is a price. J. Legal Stud..

[30] Heinelt H, Magnier A, Cabria M, Reynaert H (2018). Political Leaders and Changing Local Democracy, The European Mayor, Cham.

[31] Lenz A, Eckhard S (2022). Conceptualizing and explaining flexibility in administrative crisis management: A cross-district analysis in Germany. J. Public Administ. Res. Theory.

[32] Creemers, R. China’s social credit system: An evolving practice of control (2021). Accessed at 10.2139/ssrn.3175792 on October 17, 2022.

[33] US Congress, H.R.3684 - Infrastructure Investment and Jobs Act (2022). Accessed at https://www.congress.gov/bill/117th-congress/house-bill/3684/text on December 29, 2022.

[34] German Federal Ministry of Finance (2021). 28th Subsidy Report: Federal Government Report on Trends in Federal Financial Assistance and Tax Benefits for the Years 2019 to 2022.

[35] Acharya B, Dhakal C (2021). Implementation of state vaccine incentive lottery programs and uptake of COVID-19 vaccinations in the United States. JAMA Network Open.

[36] Office of Evaluation Science, Understanding and improving how policymakers respond to program impact. Accessed at https://oes.gsa.gov/assets/abstracts/2101-abstract-gsa-policymakers.pdf on October 17, 2022.

[37] Vivalt, E. & Coville, A. How do policy-makers update their beliefs? http://evavivalt.com/wp-content/uploads/How-Do-Policymakers-Update.pdf Accessed 17 October 2022.

[38] Vivalt, E., Coville, A. & Sampada, K. C. Weighing the evidence: Which studies count? http://evavivalt.com/wp-content/uploads/Weighing-the-Evidence.pdf Accessed 17 October 2022.

[39] Baekgaard M, Christensen J, Dahlmann CM, Mathiasen A, Petersen NBG (2019). The role of evidence in politics: Motivated reasoning and persuasion among politicians. Br. J. Polit. Sci..

[40] Lee N, Nyhan B, Reifler J, Flynn DJ (2021). More accurate, but no less polarized: Comparing the factual beliefs of government officials and the public. Br. J. Polit. Sci..

[41] DellaVigna, S., Kim, W. & Linos, E. Bottlenecks for evidence adoption. https://www.nber.org/system/files/workingpapers/w30144/w30144.pdf Accessed 17 October 2022. 10.1086/729447 PMC11449432 39372607

[42] Schneider FH, Campos-Mercade P, Meier S, Pope D, Wengström E, Meier AN (2023). Financial incentives for vaccination do not have negative unintended consequences. Nature.

[43] Campos-Mercade P, Meier AN, Schneider FH, Meier S, Pope D, Wengström E (2021). Monetary incentives increase COVID-19 vaccinations. Science.

[44] Huang Y, Huang X, Yu R (2023). The effectiveness of nonfinancial interventions and monetary incentives on COVID-19 vaccination: A meta-analysis. Health Psychol..

[45] Khazanov GK, Stewart R, Pieri MF, Huang C, Robertson CT, Schaefer KA, Ko H, Fishman J (2023). The effectiveness of financial incentives for COVID-19 vaccination: A systematic review. Prevent. Med..

[46] Serra-Garcia M, Szech N (2023). Incentives and defaults can increase COVID-19 vaccine intentions and test demand. Manag. Sci..

[47] Zhang, X. Tom, L. The backfiring effects of monetary and gift incentives on COVID-19 vaccination intentions, *China Economic Review* p. 102009 (2023). 10.1016/j.chieco.2023.102009 PMC10270730 37351337

[48] Robertson, C. *et al.* Paying americans to take the vaccine—Would it help or backfire? *J. Law Biosci.* **8**, lsab027 (2021). 10.1093/jlb/lsab027 PMC8420956 34512996

[49] Singanayagam, A. *et al.* Community transmission and viral load kinetics of the SARS-CoV-2 delta (B.1.617.2) variant in vaccinated and unvaccinated individuals in the UK: a prospective, longitudinal, cohort study. *Lancet Infect. Dis.* **22**, 183–195 (2022). 10.1016/S1473-3099(21)00648-4 PMC8554486 34756186

[50] Prunas O, Warren JL, Crawford FW, Gazit S, Patalon T, Weinberger DM, Pitzer VE (2022). Vaccination with BNT162b2 reduces transmission of SARS-CoV-2 to household contacts in Israel. Science.

[51] de Gier, B. *et al.* Vaccine effectiveness against SARS-CoV-2 transmission to household contacts during dominance of delta variant (B.1.617.2), the Netherlands, August to September 2021. *Eurosurveillance* **26**, 2100977 (2021). 10.2807/1560-7917.ES.2021.26.44.2100977 PMC8569927 34738514

[52] Buttenheim AM, Asch DA (2013). Making vaccine refusal less of a free ride. Human Vaccines Immunotherapeutics.

[53] Yeh, T.-Y. & Contreras, G. P. Full vaccination against COVID-19 suppresses SARS-CoV-2 delta variant and spike gene mutation frequencies and generates purifying selection pressure, *MedRxiv* (2021).

[54] Rella SA, Kulikova YA, Dermitzakis ET, Kondrashov FA (2021). Rates of SARS-CoV-2 transmission and vaccination impact the fate of vaccine-resistant strains. Sci. Rep..

[55] Gandon S, Lion S (2022). Targeted vaccination and the speed of SARS-CoV-2 adaptation. Proc. Natl. Acad. Sci..

[56] Betsch C, Böhm R, Korn L, Holtmann C (2017). On the benefits of explaining herd immunity in vaccine advocacy. Nature Human Behav..

[57] Agranov M, Elliott M, Ortoleva P (2021). The importance of social norms against strategic effects: The case of COVID-19 vaccine uptake. Econ. Lett..

[58] Hensel L, Witte M, Caria AS, Fetzer T, Fiorin S, Götz FM, Gomez M, Haushofer J, Ivchenko A, Kraft-Todd G (2022). Global behaviors, perceptions, and the emergence of social norms at the onset of the COVID-19 pandemic. J. Econ. Behav. Organ..

[59] Walach H, Ofner M, Ruof V, Herbig M, Klement RJ (2022). Why do people consent to receiving SARS-CoV-2 vaccinations? A representative survey in Germany. BMJ Open.

[60] Cryder CE, London AJ, Volpp KG, Loewenstein G (2010). Informative inducement: Study payment as a signal of risk. Soc. Sci. Med..

[61] Böhm R, Betsch C, Litovsky Y, Sprengholz P, Brewer NT, Chapman G, Leask J, Loewenstein G, Scherzer M, Sunstein CR (2022). Crowdsourcing interventions to promote uptake of COVID-19 booster vaccines. EClinicalMedicine.

[62] Brewer NT, Buttenheim AM, Clinton CV, Mello MM, Benjamin RM, Callaghan T, Caplan A, Carpiano RM, DiResta R, Elharake JA (2022). Incentives for COVID-19 vaccination. Lancet Region. Health-Am..

[63] Lazarus JV, Romero D, Kopka CJ, Karim SA, Abu-Raddad LJ, Almeida G, Baptista-Leite R, Barocas JA, Barreto ML, Bar-Yam Y (2022). A multinational delphi consensus to end the COVID-19 public health threat. Nature.

[64] CDC, COVID-19 vaccination field guide: 12 strategies for your community (2021). Accessed at https://www.cdc.gov/vaccines/covid-19/vaccinate-with-confidence/community.html on November 30, 2022.

[65] Mankiw, N. G. Pay people to get vaccinated (2020). Accessed at https://www.nytimes.com/2020/09/09/business/pay-people-vaccine-coronavirus.html on October 17, 2022.

[66] Vavreck, L. $100 as incentive to get a shot? Experiment suggests it can pay off (2021). Accessed at https://www.nytimes.com/2021/05/04/upshot/vaccine-incentive-experiment.html?smtyp=cur&smid=tw-nythealth on October 17, 2022.

[67] Milkman, K. Hybrid hearing on “building trust and battling barriers: The urgent need to overcome vaccine hesitancy” 117th Cong. (testimony of Katherine Milkman) (2021). Accessed at http://docs.house.gov/meetings/VC/VC00/20210701/112865/HHRG-117-VC00-Wstate-MilkmanPhDK-20210701.pdf on January 10, 2024.

[68] Thirumurthy H, Milkman KL, Volpp KG, Buttenheim AM, Pope DG (2022). Association between statewide financial incentive programs and COVID-19 vaccination rates. PloS One.

[69] Milkman, K. L. *et al.* A citywide experiment testing the impact of geographically targeted, high-pay-off vaccine lotteries, *Nature Human Behaviour* (2022). 10.1038/s41562-022-01437-0 36050387

[70] Bertelsmann Foundation, Wegweiser Kommune (2022). Accessed at https://www.wegweiser-kommune.de on October 17, 2022.

[71] Ministry of Social Affairs, Health and Integration Baden-Wuerttemberg, Vaccination data 27.12.2020- 07.11.2021 for the counties in Baden-Wuerttemberg (2021). Accessed at http://web.archive.org/web/20211111121822/. https://web.archive.org/web/20211111121822/https:/sozialministerium.baden-wuerttemberg.de/fileadmin/redaktion/m-sm/intern/downloads/Downloads_Gesundheitsschutz/Corona_Gesamtzahl-Impfungen-Landkreise-BW.pdf on November 17, 2022.

[72] Robert Koch-Institute, Vaccination rate monitoring (2022). Accessed at https://www.rki.de/DE/Content/InfAZ/N/Neuartiges_Coronavirus/Daten/Impfquotenmonitoring.xlsx on January 10, 2024.

[73] German Federal Agency for Civic Education, *Data Report 2021* (German Federal Agency for Civic Education, Bonn, 2021).

[74] Amos C, Paswan A (2009). Getting past the trash bin: Attribution about envelope message, envelope characteristics, and intention to open direct mail. J. Mark. Commun..

[75] DellaVigna S, Pope D, Vivalt E (2019). Predict science to improve science. Science.

[76] Athey S, Wager S (2019). Estimating treatment effects with causal forests: An application. Observ. Stud..

[77] Kahneman D, Tversky A (1972). Subjective probability: A judgment of representativeness. Cogn. Psychol..

[78] Tversky A, Kahneman D (1973). Availability: A heuristic for judging frequency and probability. Cogn. Psychol..

[79] Tversky A, Kahneman D (1974). Judgment under uncertainty: Heuristics and biases: Biases in judgments reveal some heuristics of thinking under uncertainty. Science.

[80] Gigerenzer G, Gaissmaier W, Kurz-Milcke E, Schwartz LM, Woloshin S (2007). Helping doctors and patients make sense of health statistics. Psychol. Sci. Public Interest.

[81] Gigerenzer G, Hertwig R, Hoffrage U, Sedlmeier P (2008). Cognitive illusions reconsidered. Handbook Exp. Econ. results.

[82] Dancy, J. Steven Pinker, Rationality: What it is, why it seems scarce, why it matters: Allen Lane, 2021, 432 pp., ISBN: 978–0241380277. *Society* **59** (2022).

[83] Zhang S, Heck PR, Meyer MN, Chabris CF, Goldstein DG, Hofman JM (2023). An illusion of predictability in scientific results: Even experts confuse inferential uncertainty and outcome variability. Proc. Natl. Acad. Sci..

[84] Broockman, D., Kalla, J., Caballero, C. & Easton, M. Political practitioners poorly predict which messages persuade the public (2023). Accessed at 10.31219/osf.io/8un6a on January 10, 2024. 10.1073/pnas.2400076121 PMC11551421 39467135

[85] Hewitt, L. *et al*. How experiments help campaigns persuade voters: Evidence from a large archive of campaigns’ own experiments. *Am. Polit. Sci. Rev.* (2024).

[86] Banerjee AV, Duflo E, Glennerster R, Kothari D (2010). Improving immunisation coverage in rural India: Clustered randomised controlled evaluation of immunisation campaigns with and without incentives. BMJ.

[87] Volpp KG, John LK, Troxel AB, Norton L, Fassbender J, Loewenstein G (2008). Financial incentive—Based approaches for weight loss: A randomized trial. JAMA.

[88] Charness G, Gneezy U (2009). Incentives to exercise. Econometrica.

[89] Avery, M., Giuntella, O. & Jiao, P. Why don’t we sleep enough? A field experiment among college students. *Rev. Econ. Stat.* 1–45 (2022).

[90] Stone EG, Morton SC, Hulscher ME, Maglione MA, Roth EA, Grimshaw JM, Mittman BS, Rubenstein LV, Rubenstein LZ, Shekelle PG (2002). Interventions that increase use of adult immunization and cancer screening services: A meta-analysis. Ann. Internal Med..

[91] Nowak MA (2006). Five rules for the evolution of cooperation. Science.

[92] Boyd R (2017). A Different Kind of Animal: How Culture Transformed Our Species.

[93] Henrich J, Muthukrishna M (2021). The origins and psychology of human cooperation. Annu. Rev. Psychol..

[94] Riggieri, A. The impact of hybrid electric vehicles incentives on demand and the determinants of hybrid-vehicle adoption. https://smartech.gatech.edu/bitstream/handle/1853/41222/RiggieriAlison201108phd.pdf (2011). Accessed 19 October 2022.

[95] Whitehead J, Washington SP, Franklin JP (2019). The impact of different incentive policies on hybrid electric vehicle demand and price: An international comparison. World Electric Vehicle J..

[96] Alberini A, Gans W, Towe C (2016). Free riding, upsizing, and energy efficiency incentives in Maryland homes. Energy J..

