# Supplementary material for: Policy makers believe money motivates more than it does
Source: Sci Rep. 2024 Jan 22;14:1901. doi: 10.1038/s41598-024-51590-x (PMC10803740; doi:10.1038/s41598-024-51590-x)
Supplement: Supplementary file 1 — Supplementary Information. [file 41598_2024_51590_MOESM1_ESM.pdf]

Supplementary Materials for

Policy makers believe money motivates more than it does

Sebastian Jilke*, Florian Keppeler, John Ternovski, Dominik Vogel, Erez Yoeli

*Correspondence to: sebastian.jilke@georgetown.edu

List of Contents

Section 1: Field Experiment **3**
1.1 Policy Context 3
1.2 Materials and Methods 5
1.3 Treatment and Control Letters 7
1.3.1 Control Letter (English Translation) 7
1.3.2 Treatment Letter (English Translation) 8
1.3.3 Control Letter (Original) 9
1.3.4 Treatment Letter (Original) 10
1.4 Data Collection 11
1.5 Additional Analyses 12
1.5.1 Descriptive Statistics 12
1.5.2 Treatment Effect by Vaccination Type 13
1.5.3 Treatment Effect Heterogeneity 13
1.5.3.1 Interaction Effects 13
1.5.3.2 Causal Forests 14
1.6 Robustness Checks 15
1.7 Pre-registered Analysis Plan 17
1.8 Data Availability 17
1.9 Statistical Power 18
1.10 Limitations 18
Section 2: Policy Makers' Knowledge and Predictions **19**
2.1 Systematic Literature Review 19
2.2 Methods and Materials 19
2.3 Additional Analyses 20
2.4 Respondent Characteristics 22
2.4.1 Prediction Heterogeneity 22
2.5 Investigating Policy Maker Non-Response 23
2.6 Survey Instrument for Policy Maker Predictions 24
Section 3: References **29**

Section 1: Field Experiment

1.1 Policy Context

The focus of this study was the entire resident population of the German city of Ravensburg. In Germany, all adult residents of the city are legally required to have a name and address record in the city's official registry. So, as part of this study, all adult residents in the city's registry received an official letter from the city government with information about seven public COVID-19 vaccination events organized by the city. No appointment was needed, and, as is the case for all of Germany, the COVID-19 vaccinations at these events were free of charge.

At the start of the vaccination campaign (November 5th, 2021), 61% of residents in Ravensburg and the surrounding county had received the first and second dose of the vaccine. This was below the average vaccination rate (62.5%) in the corresponding region, Baden-Wuerttemberg (*1*). The 7-days incidence rate per 100,000 residents was approximately 275, above the average (211) and among the highest in the region (*2*). On the first day of the vaccination campaign (11/5/2021), the German federal public health ministry officially amended its prior recommendation of booster vaccinations only for older and at-risk individuals and, instead, encouraged all German residents to get a booster dose (*3*).

Due to German laws regulating the privacy of official medical records, it is not possible to track every resident's vaccination status. As such, by documenting vaccination at public events, we have incomplete outcome data. While we can ascertain a participant was vaccinated at one of the participating events (administration of the vaccine was directly observed), we cannot ascertain that a non-attendee didn't get vaccinated elsewhere (e.g., in another city). However, since the letters sent to both treatment and control addresses advertised only a set of public events, it is reasonable to assume that any impacts of treatment would predominantly affect vaccination rates at events that participants are informed about. In Section 1.10, we address this in more detail.

Since we anticipated official government announcements during the course of our study, we pre-registered that we would present our treatment effects by event, by day, and cumulatively. The following official government announcements were recorded since the start of the campaign on November 5th, 2021 (*4*):

11/05/2021	The federal health ministry as well as the state ministries in Germany recommend booster vaccination for all persons older than 18.
11/10/2021	Federal and state authorities decide to offer only the Moderna vaccine to persons aged 30 and older, allotting the Pfizer/BioNTech vaccine for younger persons. (There were very few doses of the Pfizer/BioNTech vaccine available in Germany, at this time.)
11/17/2021	The regional health ministry of Baden-Wuerttemberg introduces the "2G rule," restricting access to most shops and restaurants to people who either 1) received the first and second dose or 2) recovered from COVID-19.
11/18/2021	The Standing Committee on Vaccination, an independent expert committee in Germany, recommends booster vaccination for all persons older than 18, six months after the second dose.
11/22/2021	The federal health ministry restricts the delivery of the BioNtech/Pfizer vaccine to

reserve it for minors under the age of 18 and offers Moderna vaccines as an alternative.

11/26/2021 The German air force starts to transport patients to other parts of the country and neighboring countries due to overcrowded hospitals.

11/27/2021 The federal health ministry officially confirms the first cases of Omicron in Germany and further restricts travel from other countries.

11/30/2021 Chancellor Scholz and the heads of German state governments announce that the 2G rule has become the nationwide standard and confirm plans for a vaccination mandate.

12/04/2021 The regional health ministry of Baden-Wuerttemberg introduces the “2G+ rule”, restricting access to most shops and restaurants to people who either 1) received the first and second dose or 2) recovered from COVID-19 and can provide a recent negative test.

12/08/2021 The state of Baden-Wuerttemberg opens further vaccination centers exclusively for booster vaccinations.

12/21/2021 The national government and the heads of German state governments announce stricter social restrictions. Starting on 12/28/2021, private gatherings of vaccinated and recovered persons are restricted to a maximum of ten persons; private gatherings of unvaccinated persons are limited to the person's own household and a maximum of two persons from another household. The Standing Committee on Vaccination recommends booster vaccinations for all persons older than 18 years of age, three months after the second dose.

1.2 Materials and Methods

The initial aim of the study was to assess the efficacy of a set of monetary incentives on people's vaccination uptake. In this manuscript, we focus on the direct effect of the financial incentive. The spillover analysis is the subject of another manuscript.

The study was conducted among the full adult population of the city of Ravensburg, Germany. All registered residents of the city (age 18 years and older) were included in the trial. As described in greater detail in our pre-registration plan, the only exclusions were the inmates of a local prison (n=257), the second largest cluster of the city (n=75), and one single-person household in a block size of one. The final study population thus included 41,548 residents nested in 10,032 address-clusters.

All registered residents of the City received a mailer in the first week of November 2021 informing them about seven public vaccination events that took place between November 13 and December 12, 2021. The letter was sent by city officials and signed by the Mayor of the city. The treatment group received a letter that included a monetary incentive of approximately $40 (i.e., 40 Euros). The incentive included an individual-level incentive of ~$20 (20 Euros) for getting vaccinated. In addition to that, a financial incentive of an additional ~$20 if more than 900 residents receive vaccinations during the seven public vaccination events was added to the letters. If recipients were already fully vaccinated (i.e., had all the recommended doses of the COVID-19 vaccine), they could still receive the community-based financial incentive if they gave their letter to another resident of the City and that other resident presented that letter while getting vaccinated at the vaccination event. The control group received the same letter as the treatment group but without the monetary incentive. A copy of the German and English-translation of both letters can be found in Section 1.3.

Treatment assignment followed a two-stage randomization process. Randomization was conducted in R (version 4.1.1) using the function "sample" with the seed set to 2021. In the first stage, all housing addresses within the city were cluster-randomized into two groups (i.e., the intervention group and the control group). The cluster-level randomization was blocked by cluster size (four address-clusters per block), to account for unequal cluster sizes (*5*). In the second stage, one resident of each address-cluster in both groups was randomly selected (a "cluster representative"). The cluster representatives in the intervention group received the intervention letter. All remaining residents were sent the control letter.

Two primary outcome measures were collected, information and vaccination uptake. In the present study, we focus on vaccination uptake only, but report the full results (which include information uptake) in a separate paper.

During the seven public vaccination events, on-site records of administered vaccinations were recorded and linked back to administrative records of the city. The vaccination events took place during the following dates: 11/13/2021, 11/19/2021, 11/20/2021, 11/26/2021, 12/10/2021 and 12/11/2021.

For the analysis, all residents living at the same address were treated as members of the same social network. As specified in our pre-analysis plan, our main estimator was a demographic covariate-adjusted OLS regression with block fixed effects and clustered standard errors (i.e., the overall effect).

For robustness, we re-ran all analyses using the pre-registered direct effect estimator and also a naive estimator that ignored household structure (i.e., the outcomes of those who received the treatment compared to all those who did not). Additionally, we used randomization inference (*6*) as an alternative means to calculate p-values. The results of these robustness checks were highly similar to that of our overall effect estimator; for more details, please. see Section 1.6.

1.3 Treatment and Control Letters

1.3.1 Control Letter (English Translation)

[Personalized Address]

Get your COVID-19 vaccination now!

Hello [ACADEMIC TITLE] [NAME],

We are contacting you today for your coronavirus vaccination.
Are you not yet fully vaccinated? Then please go for vaccination on one of the following dates:

- **13.11.:** Saturday, 9 a.m.-2 p.m.
- **19-20.11.:** Friday, 3-9 p.m. or Saturday, 9 a.m.-2 p.m.
- **26-27.11.:** Friday, 3-9 p.m. or Saturday, 9 a.m.-2 p.m.
- **10-11.12.:** Friday, 3-9 p.m. or Saturday, 9 a.m.-2 p.m.

Place: City Hall, Marienplatz 26, 88212 Ravensburg **(without appointment)**

Are you already fully vaccinated? Then motivate your environment from Ravensburg now to a vaccination on one of the mentioned dates.

Available are vaccines from BioNTech/Pfizer, Moderna and Johnson & Johnson. Please bring, if available, vaccination certificate, identity card and insurance card of the health insurance. For more information, please visit the following website: [INDIVIDUAL LINK].

Kind regards

[Signature, name, and picture of the local Mayor, chairman of the local association of the medical profession, leading physician of the local public clinic]

[Text on the right side in the column: Contact details of the sender, i.e. the Mayor, date, reference number, phone number and email address for questions, text: “all info via the link or directly via the QR code”, translation note for the languages “English, Spanish, Russian, Arabic, Turkish”, additional note: “This vaccination campaign is scientifically accompanied. It is supported by:”, logos of sponsors]

1.3.2 Treatment Letter (English Translation)

[Personalized Address]

Get your COVID-19 vaccination now, receive a 20 Euro voucher and secure the chance to win another 20 Euro!

Hello [ACADEMIC TITLE] [NAME],

Congratulations! The city of Ravensburg is giving away shopping vouchers. You are one of 5,000 **randomly selected** people from Ravensburg who can receive these limited vouchers.

Then please go for vaccination on one of the following dates.

Are you not yet fully vaccinated? Then please go for vaccination on one of the following dates. **You will receive your 20 Euros voucher for this** by mail after the vaccination.

- **13.11.:** Saturday, 9 a.m.-2 p.m.
- **19-20.11.:** Friday, 3-9 p.m. or Saturday, 9 a.m.-2 p.m.
- **26-27.11.:** Friday, 3-9 p.m. or Saturday, 9 a.m.-2 p.m.
- **10-11.12.:** Friday, 3-9 p.m. or Saturday, 9 a.m.-2 p.m.

Place: City Hall, Marienplatz 26, 88212 Ravensburg **(without appointment)**

After your vaccination on one of these dates, you also have a **chance to win**: if more than 900 people from Ravensburg get vaccinated on these dates, you will receive **your 2nd voucher worth 20 Euros** by mail.

Are you already fully vaccinated? Then motivate your environment from Ravensburg now to a vaccination on one of the mentioned dates.If this person shows your letter at one of the mentioned dates, you secure your **chance to win**: If more than 900 people from Ravensburg get vaccinated, you will receive **your 20 Euros voucher** by mail.

Available are vaccines from BioNTech/Pfizer, Moderna and Johnson & Johnson. Please bring, if available, vaccination certificate, identity card and insurance card of the health insurance. For more information, please visit the following website: [INDIVIDUAL LINK].

Kind regards

[The rest of the text is identical to the control letter.]

1.3.3 Control Letter (Original)

Jetzt Ihre COVID-19 Impfung bekommen!

Stadt Ravensburg

Stadtverwaltung · Postfach 21 80 · 88191 Ravensburg

[Personal postal address of the recipient]

[Letterhead of the Mayor]

5.11.2021
Zeichen: TEST2

Für Rückfragen:
✆ 0751 82-780
✉ mail@ravensburg.de

Alle Infos über den Link oder direkt über den QR-Code:

Jetzt Ihre COVID-19 Impfung bekommen!

[Personalized salutation]

wir kontaktieren Sie heute zur Schutzimpfung gegen das Coronavirus.

Sie sind noch nicht vollständig geimpft? Dann gehen Sie bitte an einem der folgenden Termine zur Impfung:

- **13.11.:** Samstag, 9–14 Uhr
- **19.–20.11.:** Freitag, 15–21 Uhr oder Samstag, 9–14 Uhr
- **26.–27.11.:** Freitag, 15–21 Uhr oder Samstag, 9–14 Uhr
- **10.–11.12.:** Freitag, 15–21 Uhr oder Samstag, 9–14 Uhr

Ort: Rathaus, Marienplatz 26, 88212 Ravensburg **(ohne Anmeldung)**

Sie sind bereits vollständig geimpft? Dann motivieren Sie jetzt Ihr Umfeld aus Ravensburg zu einer Impfung an einem der genannten Termine.

Verfügbar sind die Impfstoffe von BioNTech/Pfizer, Moderna und Johnson & Johnson. Bitte bringen Sie, falls vorhanden, Impfpass, Personalausweis und Versichertenkarte der Krankenkasse mit. Mehr Informationen erhalten Sie auf folgender Webseite: **https://impfkampagne.ravensburg.de/TEST2**

Freundliche Grüße

The website offers all information in other languages: English, Español, Русский, العربية, Türkçe

Diese Impfaktion wird wissenschaftlich begleitet. Sie wird unterstützt von:

GnW Gemeinsam neue Wege

MVZ Labor Ravensburg
Labor Dr. Gärtner

wifo PRO RAVENSBURG

Dr. Full Name
Mayor of the City of Ravensburg

Dr. med. Full Name
GP, Chairman of the County Medical Association

PD. Dr. med. Full Name
Chief physician at the local clinic

1.3.4 Treatment Letter (Original)

Jetzt Ihre COVID-19 Impfung bekommen und 20 Euro-Gutschein sichern!

Stadt Ravensburg

Stadtverwaltung · Postfach 21 80 · 88191 Ravensburg

[Personal postal address of the recipient]

[Letterhead of the Mayor]

5.11.2021
Zeichen: TEST1

Für Rückfragen:
✆ 0751 82-780
✉ mail@ravensburg.de

Alle Infos über den Link oder direkt über den QR-Code:

The website offers all information in other languages: English, Español, Русский, العربية, Türkçe

Diese Impfaktion wird wissenschaftlich begleitet. Sie wird unterstützt von:

GnW Gemeinsam neue Wege

MVZ Labor Ravensburg Labor Dr. Gärtner

wifo PRO RAVENSBURG

Jetzt Ihre COVID-19 Impfung bekommen, einen 20 Euro-Gutschein erhalten und die Gewinnchance auf weitere 20 Euro sichern!

[Personalized salutation]

Herzlichen Glückwunsch! Die Stadt Ravensburg verlost Einkaufsgutscheine. Sie gehören zu 5.000 **zufällig** ausgewählten Personen aus Ravensburg, die diese limitierten Gutscheine erhalten können.

Sie sind noch nicht vollständig geimpft? Dann gehen Sie bitte an einem der folgenden Termine zur Impfung. **Sie erhalten dafür nach der Impfung Ihren 20 Euro-Gutschein** per Post.

- **13.11.:** Samstag, 9–14 Uhr
- **19.–20.11.:** Freitag, 15–21 Uhr oder Samstag, 9–14 Uhr
- **26.–27.11.:** Freitag, 15–21 Uhr oder Samstag, 9–14 Uhr
- **10.–11.12.:** Freitag, 15–21 Uhr oder Samstag, 9–14 Uhr

Ort: Rathaus, Marienplatz 26, 88212 Ravensburg **(ohne Anmeldung)**

Nach Ihrer Impfung an einem dieser Termine haben Sie zudem eine **Gewinnchance**: Wenn sich mehr als 900 Personen aus Ravensburg an diesen Terminen impfen lassen, erhalten Sie **Ihren 2. Gutschein im Wert von 20 Euro** per Post.

Sie sind bereits vollständig geimpft? Dann motivieren Sie jetzt eine erwachsene Person aus Ravensburg zur Impfung. Wenn diese Person Ihr Schreiben bei einem der genannten Termine vorzeigt, sichern Sie sich die **Gewinnchance**: Wenn sich mehr als 900 Personen aus Ravensburg impfen lassen, dann erhalten Sie **Ihren 20 Euro-Gutschein** per Post.

Verfügbar sind die Impfstoffe von BioNTech/Pfizer, Moderna und Johnson & Johnson. Bitte bringen Sie, falls vorhanden, Impfpass, Personalausweis und Versichertenkarte der Krankenkasse mit. Mehr Informationen erhalten Sie auf folgender Webseite: **https://impfkampagne.ravensburg.de/TEST1**

Freundliche Grüße

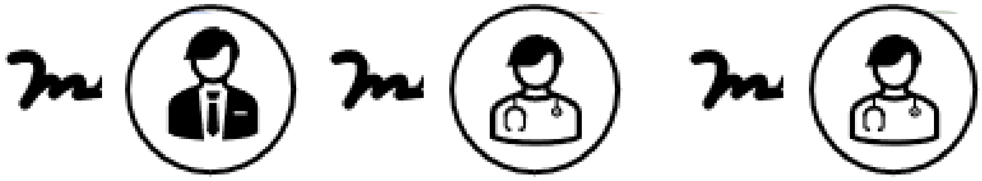

Dr. Full Name
Mayor of the City of Ravensburg

Dr. med. Full Name
GP, Chairman of the County Medical Association

PD. Dr. med. Full Name
Chief physician at the local clinic

1.4 Data Collection

In this section, we describe the data generative process underpinning our main outcome variable of interest, vaccination uptake. All outcomes were collected during the following vaccination events: 11/13/2021, 11/19/2021, 11/20/2021, 11/26/2021, 12/10/2021 and 12/11/2021.

Vaccination uptake: This variable describes whether a Ravensburg resident was vaccinated at one of the advertised vaccination events. The specific procedure for vaccination at these events is described below.

City security staff directed people to wait in line outside of the vaccination site building (to reduce the risk of infection). Before the waitlist system was implemented (see Section 1.10 for more detail), the first individual in line was invited into the vaccination site building. After the waitlist system was implemented, the individual with the announced waitlist number was invited inside. In both cases, the individual first went to the front desk where city officials collected data about the individual to be vaccinated. Representatives of the city collected the individual's full name and address from the individual's ID. This was done in accordance with the researchers' protocol and under the oversight of one of the authors. After data collection, the individual was directed to a different table in the same shared space, where a medical professional would provide a medical consultation (e.g., explain which vaccine would be provided) and administer the vaccination. The individual could still opt out after the medical consultation; **the data collection team was informed of every opt-out and flagged all opt-outs in the collected data.** (The four opt-outs had their vaccination outcome coded as zero in all of our analyses.) After vaccination, in accordance with official medical advice, the individual was directed to a monitoring room to watch for adverse (e.g., allergic) reactions to the vaccine.

Vaccination uptake was measured at each vaccination event. These data were later matched by the researchers to the list of letter recipients.

The city of Ravensburg decided to hold additional vaccination events not advertised in the letters. We analyzed this additional vaccination data in a separate paper. To check the robustness of the results reported in this paper, we re-ran all analysis with the additional outcome data and the substantive findings remained unchanged. For more details, please see (*7*).

1.5 Additional Analyses

1.5.1 Descriptive Statistics

Table S1 gives an overview of the characteristics of the participants.

Table S1. Descriptives. Counts, percentages, and means for the study's treatment arms and the total population.

	**Treatment arm**		**Control arm**		**Total**
	Cluster representatives	Cohabitants	Cluster representatives	Cohabitants	
Individuals	5,016	15,754	5,016	15,762	41,548
Clusters		5,016		5,016	10,032
Mean size of cluster		4.14		4.14	4.14
% of study population who got vaccinated	1.73%	1.80%	1.97%	2.07%	1.92%
% female of study population	51.75%	50.84%	51.54%	51.61%	51.33%
Mean Age of study population	53.22	49.03	53.06	48.85	49.96
% foreign nationals of study population	9.67%	16.65%	9.25%	17.73%	15.32%

1.5.2 Treatment Effect by Vaccination Type

Table S2. Treatment Effect by Vaccination Type. Covariate-adjusted OLS regression estimates of the effects of the treatment on all vaccinations, first dose vaccinations, primary vaccinations (first and second dose), and booster vaccinations.

	**(1)**<br>**All vaccinations**	**(2)**<br>**First dose vaccinations**	**(3)**<br>**Primary vaccinations**	**(4)**<br>**Booster vaccinations**
Treatment	-0.003 (0.001)+	-0.000 (0.001)	0.000 (0.001)	-0.003 (0.001)**
Age	0.000 (0.000)	-0.000 (0.000)	-0.000 (0.000)+	0.000 (0.000)**
Age^2	-0.000 (0.000)	-0.000 (0.000)	0.000 (0.000)	-0.000 (0.000)
Female	0.000 (0.001)	-0.002 (0.001)*	-0.002 (0.001)*	0.002 (0.001)*
Non-German	0.010 (0.003)***	0.012 (0.002)***	0.015 (0.002)***	-0.004 (0.001)**
Num.Obs.	41,548	40,998	41,059	41,241
R2	0.082	0.060	0.066	0.096
R2 Adj.	0.023	-0.001	0.005	0.037
R2 Within	0.001	0.004	0.005	0.002
R2 Within Adj.	0.001	0.004	0.005	0.002
AIC	-45757.5	-91165.7	-82571.2	-65491.0
BIC	-24058.7	-69500.4	-60902.2	-43810.9
RMSE	0.13	0.07	0.08	0.10

Standard errors (clustered by address clusters) in parentheses.
+ $p < 0.1$, * $p < 0.05$, ** $p < 0.01$, *** $p < 0.001$.

1.5.3 Treatment Effect Heterogeneity

We investigate treatment effect heterogeneity in two ways: 1) through a series of regressions that interact the treatment indicator against each available covariate, and 2) causal forests.

1.5.3.1 Interaction Effects

As seen via the interaction terms in Table S3, treatment effects did not significantly differ across age, gender, and nationality. The same lack of heterogeneity was found in terms of impact on booster vaccinations and is discussed in more detail in a separate paper.

Table S3. Covariate-adjusted OLS regression estimates of the interaction of the treatment with age, gender, and nationality.

	(1)<br>**Main effect**	(2)<br>**Age**	(3)<br>**Gender**	(4)<br>**Nationality**
Treatment	-0.003 (0.001)+	-0.002 (0.004)	-0.001 (0.002)	-0.002 (0.001)+
Age	0.000 (0.000)	0.000 (0.000)	0.000 (0.000)	0.000 (0.000)
Age^2	-0.000 (0.000)	-0.000 (0.000)	-0.000 (0.000)	-0.000 (0.000)
Female	0.000 (0.001)	0.000 (0.001)	0.002 (0.002)	0.000 (0.001)
Non-German	0.010 (0.003)***	0.010 (0.003)***	0.010 (0.003)***	0.011 (0.004)*
Treatment x Age		-0.000 (0.000)		
Treatment x Female			-0.002 (0.003)	
Treatment x Non-German				-0.001 (0.006)
Num.Obs.	41,548	41,548	41,548	41,548
R2	0.082	0.082	0.082	0.082
R2 Adj.	0.023	0.023	0.023	0.023
R2 Within	0.001	0.001	0.001	0.001
R2 Within Adj.	0.001	0.001	0.001	0.001
AIC	-45757.5	-45755.5	-45756.2	-45755.5
BIC	-24058.7	-24048.1	-24048.8	-24048.1
RMSE	0.13	0.13	0.13	0.13

Standard errors (clustered by address clusters) in parentheses.
+ $p < 0.1$, * $p < 0.05$, ** $p < 0.01$, *** $p < 0.001$.

1.5.3.2 Causal Forests

To assess treatment effect heterogeneity, we use causal forests (*8*, *9*) to predict individual-level treatment effects. The predictors in the model were participants' age, gender, nationality (German vs. Non-German), the size of the address cluster, and the mean distance between the

address and the two vaccination sites as predictors. This analysis was conducted using the grf package in *R* (*10*) with parameter-tuning.

We use augmented inverse-propensity weighting to estimate a null average treatment effect -0.27 percentage points (SE = 0.17 percentage points). Additionally, we find no evidence of treatment effect heterogeneity; an omnibus significance test fails to reject the null hypothesis of homogeneity at p-value = 0.93. Table S4 depicts the relative importance of the predictors for explaining the minor, non-significant variations in treatment effects across individuals.

Table S4. Relative importance of predictors for treatment effect heterogeneity. Causal forest estimation.

**Predictor**	**Importance**
Age	0.267
Female	0.046
Non-German	0.060
Address cluster size	0.257
Distance to vaccination site	0.370

1.6 Robustness Checks

We present the results of three different treatment effect estimators in Table S5 below. The first row in Column 1 shows the covariate-adjusted difference in vaccination rate between treatment and control for our **entire universe** (i.e., the average vaccination rate among individuals who received the treatment letter vs. the average vaccination rate among individuals who received the control letter). This estimate makes use of all of the data in our RCT and assumes there are no spillover effects. This is the result we report in the main article. The first row in Column 2 shows the covariate-adjusted difference in vaccination rates for only the randomly selected **cluster-address representatives** (i.e., cluster-representatives who received the treatment letter and cluster-representatives who received the control). This estimate isolates the direct effect of the treatment letter and guards against possible spillover effects. The first row in Column 3 shows the covariate-adjusted difference in vaccination rate for **households** (i.e., the average vaccination rate in address-clusters that received the treatment letter vs. the average vaccination rate in address-clusters that received only control letters). This estimate includes complex spillover effects within households. And while only the estimators in columns 2-3 were pre-registered, the three estimators produce nearly identical treatment effect estimates in all reported analyses.

Table S5. Estimation of three possible treatment effects. Covariate-adjusted OLS regression.

	(1) Overall effect	(2) Direct effect	(3) Treatment vs. control group
Treatment	-0.003 (0.001)+	-0.002 (0.003)	-0.001 (0.002)
Age	0.000 (0.000)	0.000 (0.000)	0.000 (0.000)
Age^2	-0.000 (0.000)	-0.000 (0.000)	-0.000 (0.000)
Female	0.000 (0.001)	0.001 (0.003)	0.000 (0.001)
Non-German	0.010 (0.003)***	0.012 (0.006)*	0.010 (0.003)***
Num.Obs.	41,548	10,032	41,548
R2	0.082	0.253	0.082
R2 Adj.	0.023	0.004	0.023
R2 Within	0.001	0.001	0.001
R2 Within Adj.	0.001	0.000	0.001
AIC	-45757.5	-9624.7	-45753.8
BIC	-24058.7	8502.9	-24055.0
RMSE	0.13	0.12	0.13
Std.Errors	clustered by address clusters	Heteroskedasticity-robust	clustered by address clusters

Standard errors in parentheses. + $p < 0.1$, * $p < 0.05$, ** $p < 0.01$, *** $p < 0.001$.

We then use randomization inference to assess how often we would recover the distribution of outcomes we see in our observed data under different conditions. We take the outcome distribution, randomly permute the treatment assignment vector, while keeping the rest of our data frame fixed, for 1,000 iterations. Comparing this synthetic null distribution gives us a standard randomization inference p-value of 0.104 for our overall treatment effect.

Next, we evaluate the impact of iteratively adding a “true” treatment effect in increments of 0.01 percentage points. In other words, all those individuals permuted to a synthetic treatment have the probability of their synthetic outcome variable increased by one percentage point. We use our main estimators to compute the treatment effect coefficient of interest. We repeat for 1,000 iterations to give us a synthetic distribution where we know the true effect. We then assess how likely our observed treatment effect estimates are under this synthetic distribution. We repeat until the probability of observing the treatment effect in our observed data is less than .05. We find that if the *true* overall effect is at least 0.003 with a constant spillover effect of 0.002, it would appear as observed in our data only 2.0% of the time.

In sum, these analyses provide robust evidence that the treatment effect of the intervention on vaccination is close to zero.

1.7 Pre-registered Analysis Plan

We pre-registered the data collection, analysis, and hypotheses at the Open Science Framework (https://osf.io/2trmb) and at ISRCTN (https://www.isrctn.com/ISRCTN59503725). **Note that this publication investigates hypothesis H3-2, the remaining analyses are addressed in a forthcoming paper *(7)*.**

1.8 Data Availability

Since our data contains sensitive health information and the associated socio-demographics could be used to de-anonymize individuals, we cannot provide the full data publicly. Instead, we provide a reduced dataset containing the outcome variable (vaccination uptake), treatment condition (treatment arm and cluster representative status), and cluster ID. To increase anonymity, the data was salted by randomly selecting three vaccinated individuals and inverting their vaccination outcome status, and three unvaccinated individuals and inverting their status. This was done four times: in the treatment arm and in the control arm among address clusters with 25 or more cohabitants, and in the treatment and control arm among clusters with less than 25 cohabitants. Hence, the vaccination outcome for 24 individuals has been changed. This enables interested researchers to reproduce the main results without adjusting for covariates (which do not alter the results) while maintaining the anonymity of all participants. Additionally, we provide all R analysis code and its full output in a separate document.

1.9 Statistical Power

The code used for power calculations can be found here: https://doi.org/10.17605/OSF.IO/UH2SD. We created a synthetic dataset using binomial distributions to determine the control and treatment potential outcomes. To take into account the presence of fully vaccinated individuals, we first drew from a binomial distribution with a base rate of 20%. These 20% would always appear as zeros for all potential outcomes. Then we generated different potential outcomes depending on whether the individual received the treatment letter (for direct effect power), are cohabitants with someone who received the treatment letter (for spillover effect power), and an aggregate effect that ignores the possibility of differing direct and spillover effects. We randomly generate treatment conditions and determine if our estimator recovers significant treatment effects over 500 such iterations.

Based on these simulation-based power calculations, we are able to detect a minimal direct effect of 2.6 percentage points, a spillover effect of 1.5 percentage points, and an overall effect of 1.3 percentage points with approximately 80% statistical power (alpha = 0.05). We projected that up to 20% of the control group will get vaccinated at these events.

1.10 Limitations

As mentioned in Section 1.1, it is not possible to have complete vaccination information for all Ravensburg residents in our sample. We only tracked vaccinations at the city-organized public events. We investigated the possibility of displacement of vaccinations and effects of wait lines in a separate paper.

The first three events had more demand than the city anticipated, which resulted in long lines and some latecomers being turned away due to a limited availability of doses. For additional information, please see the coverage in the regional German newspaper, Schwaebische Zeitung (*11*).

To ensure that media reporting did not compromise our experimental design, the Mayor of Ravensburg reached out to local media ahead of time and asked news outlets to delay coverage of the monetary incentives until after the experiment. We monitored local and national media outlets throughout the experiment and found that there was no press coverage of the financial incentives.

Photographs illustrating the vaccination events and the long lines outside can be provided by the authors upon request.

Section 2: Policy Makers' Knowledge and Predictions

2.1 Systematic Literature Review

A systematic review of existing research yielded no studies that discuss policy makers' knowledge about the efficacy of monetary incentives. We searched the database, Web of Science, to identify English-language, peer-reviewed, scientific publications (published before 12/31/2022) based on the following search query:

"((ALL=((policy*maker* OR government executive* OR mayor* OR politician*))) AND ALL=((attitude* OR perspective* OR view* OR perception* OR perceive* OR think))) AND ALL=(((financial OR monetary) AND (incentive* OR reward* OR stimul* OR compensation)))"

This search yielded 483 publications. We used the AI-based ASReview tool (v.1.1; *12*, *13*) to screen the title and abstract of each publication. We then manually coded 15.5% (i.e., 75) of title-abstract-combinations as to whether they discussed policy makers' knowledge of the efficacy of monetary incentives. On this basis, the trained automation tool ranked the remaining studies by their relevance. As outlined, we could not identify a single study that empirically assessed policy makers' knowledge on the efficacy of monetary incentives.

2.2 Methods and Materials

We conducted an online survey to collect policy makers' predictions of the impact of our treatment on vaccination uptake. We emailed 815 mayors of German cities with more than 30,000 inhabitants and heads of German counties. Around 10% of respondents reported that their locality had less than 30,000 residents. This could be that some of them were asked to fulfill the tasks of a district or they were encouraged to participate by the mayor of Ravensburg. 81 emails bounced back because they were no longer valid. 88 policy makers participated in the survey (44% of them were experts within the administration who were forwarded the survey email by their mayor/county head).

In the survey, the policy makers were presented with a description of our experiment and a copy of the treatment letter. Subsequently, they were asked to forecast what proportion of those who received a letter with and without a monetary incentive would get vaccinated. Additionally, we asked them to predict the vaccination rate for those who did not get a monetary incentive but lived in a household with somebody who did get a monetary incentive, as well as the vaccination uptake of households where nobody received a monetary incentive.

This within-person design necessitated exposing our respondents to both the letter with a monetary incentive and the letter without. This design may be vulnerable to demand effect bias and other related cognitive biases, which may induce a participant to predict larger differences between two choices (over no differences). An alternative option to presenting policy makers would be a between-subjects design. However, we felt a between-subjects survey was intractable

due to power considerations. For instance, our within-subjects design is well-powered, even without control covariates, to detect effects as small as 4.6 percentage points. Assuming similar variances in a between-subjects design, we would need 670 respondents (or a, likely unattainable, 82% survey completion rate).

A within-subjects design is also more aligned with the decision-making environment a policy maker is likely to find themselves in (choosing between two options, a letter with incentives, or one without them), rather than two policy makers in different municipalities choosing whether to adopt a letter with no knowledge of the alternatives. Research design handbooks predominantly caution about three key mechanisms that could distort treatment effect estimates in within-subject designs: practice/learning, sensitization, and carryover (e.g., *14*, *15*) - all of which are not applicable in our policy choice context. Indeed, within-subject designs appear to be standard practice among other expert surveys (*16*, *17*, *18*).

2.3 Additional Analyses

Fig. S1. Policy makers' predictions as percentage differences between treatment and control group.

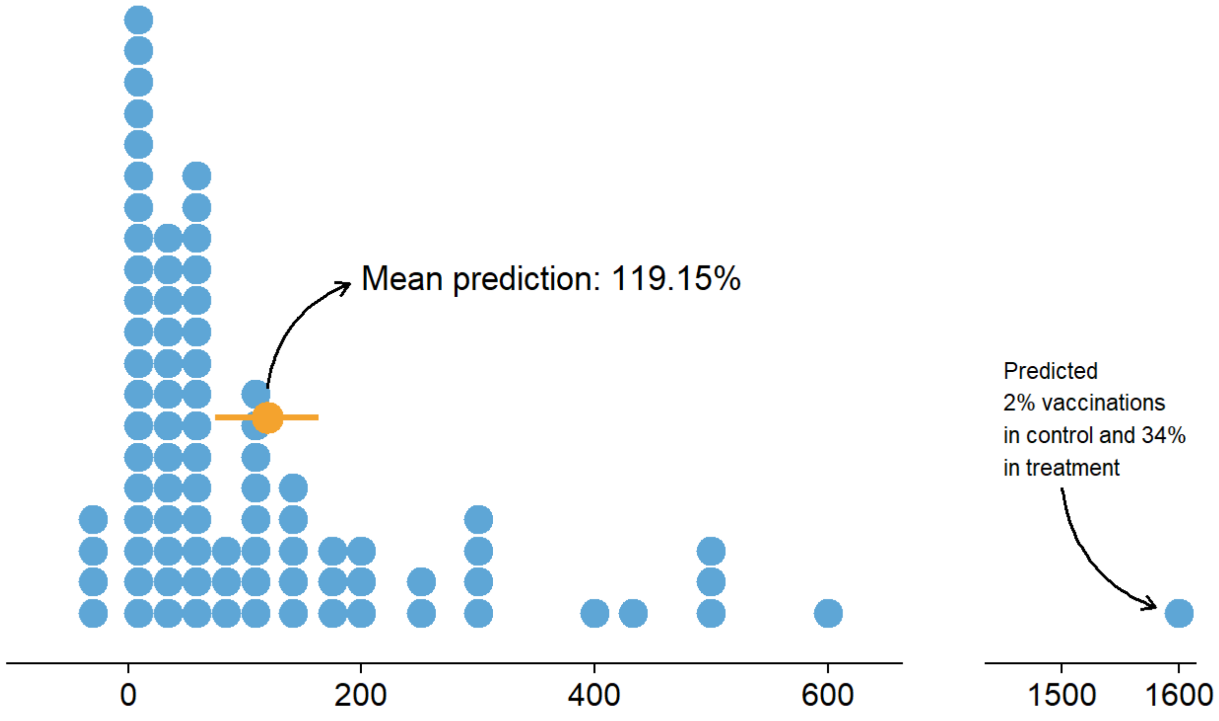

Overall, policy makers predicted a high proportion of vaccinations. They predicted, on average, that 27.5% (*SD* = 20.1) of people in the no-incentive condition would get vaccinated and 42.8% (*SD* = 24.0) in the monetary incentive condition would get vaccinated (see Fig. S2). In other words, the participating policy makers estimated a positive treatment effect of 15.3 percentage points (*SD* = 15.7, *t*(86)=-9.06, *p*<.001).

Fig. S2. Policy makers' predictions of vaccine uptake by individuals who did or did not receive a monetary incentive

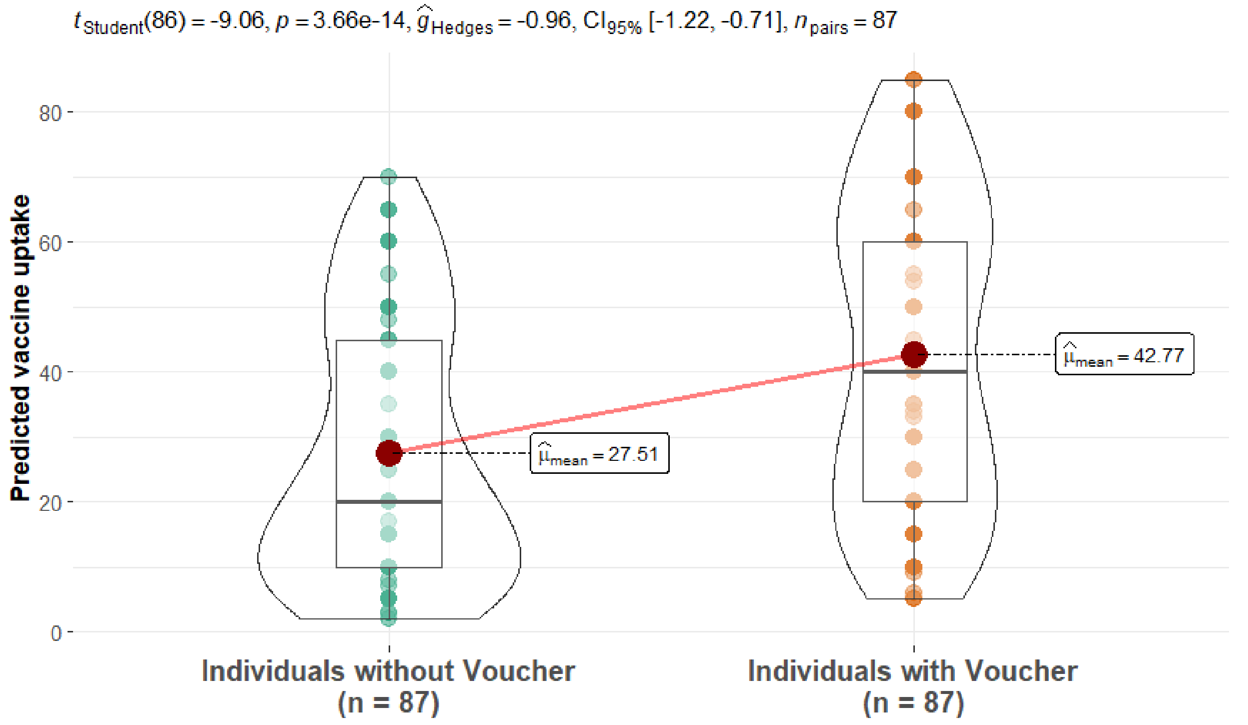

It should be noted, for the sake of transparency, that the policy maker survey was conducted in March 2022, after the field experiment was completed in December 2021. However, the results of the field experiment were not shared with anyone outside of the co-authors of this manuscript. In other words, even collaborators in the Ravensburg city administration were not informed of the results.

2.4 Respondent Characteristics

Table S5. Policy Maker Characteristics.

Survey Respondents	N = 88
**Number of Residents in the Locality the Respondent Presides Over**	**n (%)**
<= 50,000	28 (31.8%)
50,000-100,000	20 (22.7%)
> 100,000	39 (44.3%)
did not respond to this question	1 (1.1%)
**Experience in current position**	
up to 5 years	49 (55.7%)
more than 5 years	39 (44.3%)
**Position**	
(Lord) Mayor / District head	49 (55.7%)
Department Head / Other positions	39 (44.3%)

2.4.1 Prediction Heterogeneity

Below, we present a series of subgroup analyses across participant characteristics. We find that the policy makers' predictions were fairly homogenous. In all, no subgroup was statistically significantly different from any other subgroup.

Table S6. Predictions across respondent municipality size

Respondent's municipality size (in # of residents)	Predicted Impact	95% CI	*t*-Test
> 100.000	17.395	12.404, 22.386	$t(37) = 7.06$, $p < .001$
50.000-100.000	9.450	1.816, 17.084	$t(19) = 2.59$, $p = .018$
< 50.000	16.536	10.408, 22.664	$t(27) = 5.54$, $p < .001$

Table S7. Predictions across respondent experience

Respondent's Experience	Predicted Impact	95% CI	*t*-Test
Up to 5 years	16.521	11.868, 21.174	$t(47) = 7.14$, $p < .001$
More than 5 years	13.718	8.734, 18.702	$t(38) = 5.57$, $p < .001$

Table S8. Predictions across respondent position

Respondent's Position	Predicted Impact	95% CI	*t*-Test
Mayor or district head	15.55	10.78, 20.32	$t(48) = 6.55$, $p < .001$
Other	14.89	10.07, 19.72	$t(37) = 6.26$, $p < .001$

2.5 Investigating Policy Maker Non-Response

It is possible that our respondents are systematically different from non-respondents. To assess how nonresponse or selection bias may impact our results, we pursue a bounding exercise. We take all non-respondents and simulate different levels of non-response bias. Specifically, for all 727 policy makers who did not respond to our survey, we generate possible predictions. The first row in Table S9 assumes maximal nonresponse bias; in other words, we assume that the average non-respondents' prediction was equivalent to the actual results of the financial incentive RCT in Ravensburg. This is operationalized as treatment effects drawn from a normal distribution with mean equivalent to the mean treatment effect we observed in the RCT and the variance set to the Neymann variance measured in the RCT.

The first column of Table S9 depicts the simulated mean prediction among non-respondents. Since the first row assumes maximal non-response bias, it is equivalent to the null result in our RCT. We then combine the real response data from respondents with the simulated data from non-respondents and calculate the prediction we would have observed under maximal nonresponse bias. The second column depicts this result. Specifically, we see that even with maximal nonresponse bias, policy makers would be significantly (as seen from the confidence interval in column 3) misaligned on the null effect of financial incentives.

With each row, we relax the magnitude of nonresponse bias by 2 percentage points. So for row 2, policy makers who did not respond are still much closer to the null result than our respondents (only 2 percentage points off) but not as preternaturally prescient of the true impact of financial incentives as in the first row. We continue to decrease accuracy (by an iteration of 2 percentage points) until the non-respondent prediction is equal to the mean predicted impact we report in our

main text. In all, we see that our results are robust to even moderate to large levels of nonresponse bias.

Table S9. The impact of different levels of nonresponse bias on the reported results (in percentage points)

Possible non-respondent prediction	Combined (respondent & non-respondent) prediction	Combined prediction 95% CI
-0.3	1.4	0.9, 1.9
1.7	3.2	2.7, 3.6
3.7	5.0	4.5, 5.4
5.7	6.8	6.3, 7.2
7.7	8.5	8.2, 8.9
9.7	10.3	10.0, 10.7
11.7	12.1	11.8, 12.5
13.7	13.9	13.5, 14.3
15.7	15.7	15.3, 16.0

2.6 Survey Instrument for Policy Maker Predictions

Below is a condensed version of the survey instrument sent to policy makers. The full version (which includes questions on incentive spillovers) and the corresponding analyses are the subject of a separate paper.

> Dear Sir or Madam,
>
> We are contacting you today in connection with a vaccination campaign that we have implemented in partnership with the city of Ravensburg. Our project partner Simon Blümcke (First Mayor of the City of Ravensburg), supports this survey.
>
> In the wake of the ongoing Corona pandemic, we are exploring local decision-makers' attitudes toward interventions to increase vaccination preparedness. This survey aims to determine how you – at a local level – rate the effectiveness of monetary incentives as part of vaccination campaigns.

In the following, we would like to ask you some short questions about a vaccination campaign that the city of Ravensburg carried out with our scientific support. We would like to ask you to share your professional experience here. There are no right or wrong answers. The survey is completely anonymous and takes about 10 minutes.

Thank you for your support!

[NEXT PAGE]

Background:

The city of Ravensburg has approx. 50,000 inhabitants (of which approx. 41,000 are adults). The rate of persons with first and second shots in the district of Ravensburg before the start of the vaccination campaign at the end of October 2021 was around 60%. Almost no one had already had a booster vaccination at that time.

The campaign:

The vaccination campaign included seven public vaccination appointments (between 11/13/2021 and 12/11/2021), to which all adult residents were invited via mail. One could receive a first, second or booster vaccination on the vaccination dates. All adult residents received **one of two different versions of** a letter. The two versions of the same letter were sent out on 11/05/2021. Who received which letter was determined at **random**.

- **Version 1** – **With voucher:** A number of randomly selected adult residents (approx. 5,000) were sent the following letter from the city of Ravensburg. These residents were given the opportunity to receive a **20 Euro voucher** if they were vaccinated on one of the vaccination dates. **In addition, all recipients had the chance to receive a second 20 Euro voucher if more than 900 adults from Ravensburg were vaccinated on the dates**. Those who had already been fully vaccinated could pass the letter on to an unvaccinated person and thereby also receive the chance to win the second 20-euro voucher.
- **Version 2** – **Without voucher**: All other residents received a regular letter **without** financial incentive.

Version 1 (with voucher) of the cover letter	Version 2 (without voucher) of the cover letter

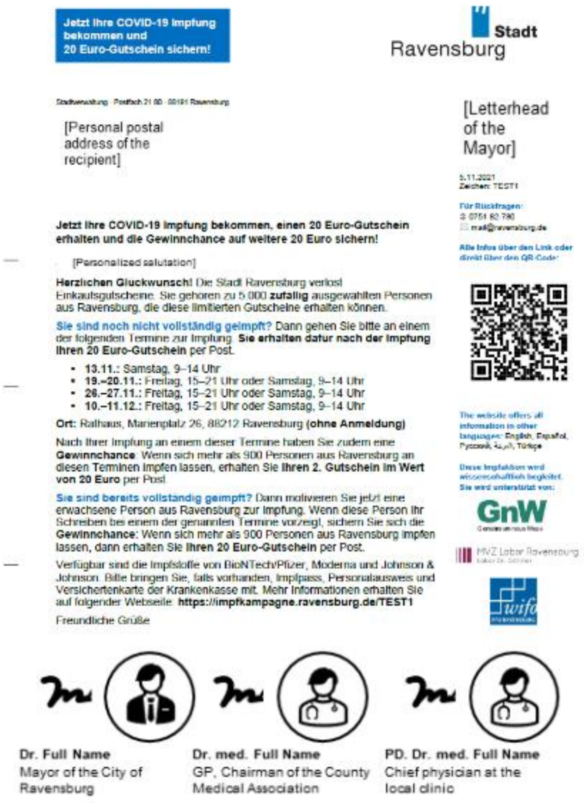

Jetzt Ihre COVID-19 Impfung bekommen und 20 Euro-Gutschein sichern!

Stadt Ravensburg

Stadtverwaltung · Postfach 21 80 · 88181 Ravensburg

[Personal postal address of the recipient]

[Letterhead of the Mayor]

5.11.2021
Zeichen: TEST1

Für Rückfragen:
0751 82 780
mail@ravensburg.de

Alle Infos über den Link oder direkt über den QR-Code:

Jetzt Ihre COVID-19 Impfung bekommen, einen 20 Euro-Gutschein erhalten und die Gewinnchance auf weitere 20 Euro sichern!

[Personalized salutation]

Herzlichen Glückwunsch! Die Stadt Ravensburg verlost Einkaufsgutscheine. Sie gehören zu 5.000 **zufällig** ausgewählten Personen aus Ravensburg, die diese limitierten Gutscheine erhalten können.

Sie sind noch nicht vollständig geimpft? Dann gehen Sie bitte an einem der folgenden Termine zur Impfung. **Sie erhalten dafür nach der Impfung Ihren 20 Euro-Gutschein** per Post.

- **13.11.:** Samstag, 9–14 Uhr
- **19.–20.11.:** Freitag, 15–21 Uhr oder Samstag, 9–14 Uhr
- **26.–27.11.:** Freitag, 15–21 Uhr oder Samstag, 9–14 Uhr
- **10.–11.12.:** Freitag, 15–21 Uhr oder Samstag, 9–14 Uhr

Ort: Rathaus, Marienplatz 26, 88212 Ravensburg **(ohne Anmeldung)**

Nach Ihrer Impfung an einem dieser Termine haben Sie zudem eine **Gewinnchance**: Wenn sich mehr als 900 Personen aus Ravensburg an diesen Terminen impfen lassen, erhalten Sie **Ihren 2. Gutschein im Wert von 20 Euro** per Post.

Sie sind bereits vollständig geimpft? Dann motivieren Sie jetzt eine erwachsene Person aus Ravensburg zur Impfung. Wenn diese Person Ihr Schreiben bei einem der genannten Termine vorzeigt, sichern Sie sich die **Gewinnchance:** Wenn sich mehr als 900 Personen aus Ravensburg impfen lassen, dann erhalten Sie **Ihren 20 Euro-Gutschein** per Post.

Verfügbar sind die Impfstoffe von BioNTech/Pfizer, Moderna und Johnson & Johnson. Bitte bringen Sie, falls vorhanden, Impfpass, Personalausweis und Versichertenkarte der Krankenkasse mit. Mehr Informationen erhalten Sie auf folgender Webseite: **https://impfkampagne.ravensburg.de/TEST1**

Freundliche Grüße

The website offers all information in other languages: English, Español, Русский, العربية, Türkçe

Diese Impfaktion wird wissenschaftlich begleitet. Sie wird unterstützt von:

GnW

MVZ Labor Ravensburg

wifo

Dr. Full Name
Mayor of the City of Ravensburg

Dr. med. Full Name
GP, Chairman of the County Medical Association

PD. Dr. med. Full Name
Chief physician at the local clinic

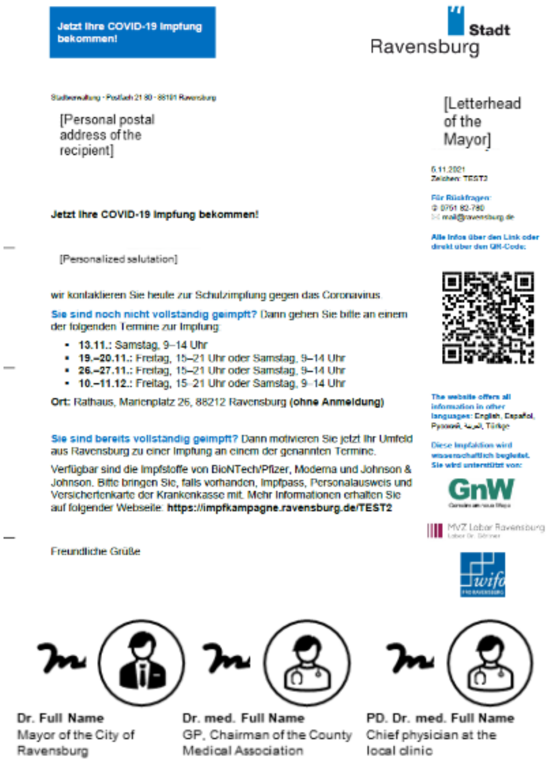

Jetzt Ihre COVID-19 Impfung bekommen!

Stadt Ravensburg

Stadtverwaltung · Postfach 21 80 · 88181 Ravensburg

[Personal postal address of the recipient]

[Letterhead of the Mayor]

5.11.2021
Zeichen: TEST2

Für Rückfragen:
0751 82-780
mail@ravensburg.de

Alle Infos über den Link oder direkt über den QR-Code:

Jetzt Ihre COVID-19 Impfung bekommen!

[Personalized salutation]

wir kontaktieren Sie heute zur Schutzimpfung gegen das Coronavirus.

Sie sind noch nicht vollständig geimpft? Dann gehen Sie bitte an einem der folgenden Termine zur Impfung:

- **13.11.:** Samstag, 9–14 Uhr
- **19.–20.11.:** Freitag, 15–21 Uhr oder Samstag, 9–14 Uhr
- **26.–27.11.:** Freitag, 15–21 Uhr oder Samstag, 9–14 Uhr
- **10.–11.12.:** Freitag, 15–21 Uhr oder Samstag, 9–14 Uhr

Ort: Rathaus, Marienplatz 26, 88212 Ravensburg **(ohne Anmeldung)**

Sie sind bereits vollständig geimpft? Dann motivieren Sie jetzt Ihr Umfeld aus Ravensburg zu einer Impfung an einem der genannten Termine.

Verfügbar sind die Impfstoffe von BioNTech/Pfizer, Moderna und Johnson & Johnson. Bitte bringen Sie, falls vorhanden, Impfpass, Personalausweis und Versichertenkarte der Krankenkasse mit. Mehr Informationen erhalten Sie auf folgender Webseite: **https://impfkampagne.ravensburg.de/TEST2**

Freundliche Grüße

The website offers all information in other languages: English, Español, Русский, العربية, Türkçe

Diese Impfaktion wird wissenschaftlich begleitet. Sie wird unterstützt von:

GnW

MVZ Labor Ravensburg

wifo

Dr. Full Name
Mayor of the City of Ravensburg

Dr. med. Full Name
GP, Chairman of the County Medical Association

PD. Dr. med. Full Name
Chief physician at the local clinic

[NEXT PAGE]

As we move forward, we would like to ask you some questions about this vaccination campaign. We would first like to ensure that we have made the details of the campaign sufficiently clear. To this end, we ask you to select below the one of the four statements that describes the said vaccination campaign as precisely as possible:

1. All Ravensburg residents were promised a 20-Euro vaccination voucher if they got vaccinated.
2. Only a few randomly selected residents were promised a 20-Euro vaccination voucher if they got vaccinated. The voucher was not transferable.
3. Only a few randomly selected residents were promised a 20-Euro vaccination voucher if they got vaccinated. In the case that more than 900 residents were vaccinated, a second voucher was promised. Those randomly

selected people who were already fully vaccinated could still receive the second voucher if they had transferred the letter to another person.

4. No vouchers were offered to the inhabitants of Ravensburg.

[Explain the design again if participants selected the wrong answer]

[NEXT PAGE]

First, we would like you to give us your *assessment* regarding **all types of** vaccinations (first, second, and booster).

Q1.1: What percentage of adult residents who received a letter **with** a voucher were vaccinated on one of the seven events (first, second, and booster vaccination)?

Your estimate: 0-100%

Q1.2: What percentage of adult residents who received a letter **without** a voucher were vaccinated on one of the seven events (first, second, and booster vaccination)?

Your estimate: 0-100%

[NEXT PAGE]

In conclusion to this survey, we would like to collect some information about you briefly:

- How many inhabitants does the local authority for which you work have?
  - Less than 10,000
  - 10.000-20.000
  - 20.001-30.000
  - 30.001-40.000
  - 40.001-50.000
  - 50.001-100.000

- More than 100,000

- How many years have you held your current position?
  - less than a year
  - 1 to 5 years
  - 6 to 10 years
  - 11 to 15 years
  - 16 to 20 years
  - More than 20 years
- What is your current position?
  - Councilor
  - Mayor
  - Lord Mayor
  - District Administrator
  - Other: _________

If you would like to share any additional information about the vaccination campaign with us, please use the following free text field:

Section 3: References

1. Ministry of Social Affairs, Health and Integration Baden-Wuerttemberg, Vaccination data 12/27/2020 - 11/07/2021 by district in Baden-Wuerttemberg (Impfdaten 27.12.2020 -

07.11.2021 nach Landkreisen in Baden-Württemberg) (2021), (available at http://web.archive.org/web/20211111121822/https:/sozialministerium.baden-wuerttemberg.de/fileadmin/redaktion/m-sm/intern/downloads/Downloads_Gesundheitsschutz/Corona_Gesamtzahl-Impfungen-Landkreise-BW.pdf).

2. State Health Department Baden-Wuerttemberg, Daily Report COVID-19 (Tagesbericht COVID-19 05.11.2021) (2021), (available at https://www.baden-wuerttemberg.de/fileadmin/redaktion/dateien/PDF/Coronainfos/211105_COVID_Tagesbericht_LGA_01.pdf).

3. Federal Ministry of Health, 94th Conference of Ministers of Health, Resolution from 05.11.2021 (94. Gesundheitsministerkonferenz, Beschluss vom 05.11.2021) (2021), (available at https://www.bundesgesundheitsministerium.de/fileadmin/Dateien/3_Downloads/G/GMK/GMK_Beschluss_05.11.2021_.pdf).

4. Central German Broadcasting (Mitteldeutscher Rundfunk), The Chronicle of the Corona Crisis 2021 (Die Chronik der Corona-Krise 2021) (2022), (available at https://www.mdr.de/nachrichten/jahresrueckblick/corona-nachrichten-jahresrueckblick-chronologie-100.html#sprung0).

5. A. S. Gerber, D. P. Green, Field Experiments: Design, Analysis, and Interpretation (Norton, New York, NY, 2012).

6. G. Imbens, D. B. Rubin, Causal Inference for Statistics, Social, and Biomedical Sciences: An Introduction (Cambridge University Press, New York, 2015).

7. J. Ternovski, S. Jilke, F. Keppeler, D. Vogel, Scaling Behavioral Interventions in the Presence of Spillover: Implications for Randomized Controlled Trials and Evidence-Based Policy Making. *Working Paper* (2023).

8. S. Athey, S. Wager, Estimating Treatment Effects with Causal Forests: An Application. *Observational Studies*. **5**, 37–51 (2019).

9. S. Athey, J. Tibshirani, S. Wager, Generalized Random Forests. *The Annals of Statistics*. **47**, 1148–1178 (2019).

10. J. Tibshirani, S. Athey, R. Friedberg, V. Hadad, D. Hirshberg, L. Miner, E. Sverdrup, S. Wager, M. Wright, grf: Generalized Random Forests (2022), (available at https://CRAN.R-project.org/package=grf).

11. S. Keppeler, Again Vaccination Willing People Storm the Free Offer in Ravensburg at the Weekend (Wieder Stürmen Impfwillige am Wochenende das freie Angebot in Ravensburg). *Schwaebische Zeitung* (2021), (available at https://www.schwaebische.de/landkreis/landkreis-ravensburg/ravensburg_artikel,-wieder-stuermen-impfwillige-am-wochenende-das-freie-angebot-in-ravensburg-_arid,11438174.html)

12. R. van de Schoot, J. de Bruin, R. Schram, P. Zahedi, J. de Boer, F. Weijdema, B. Kramer, M. Huijts, M. Hoogerwerf, G. Ferdinands, A. Harkema, J. Willemsen, Y. Ma, Q. Fang, S. Hindriks, L. Tummers, D. L. Oberski, An Open Source Machine Learning Framework for Efficient and Transparent Systematic Reviews. *Nat Mach Intell*. **3**, 125–133 (2021).

13. ASReview LAB Developers, ASReview LAB - A Tool for AI-assisted Systematic Reviews (2022), v.1.1, doi:10.5281/ZENODO.3345592.

14. G. Keren, C. Lewis, Eds., *A Handbook for Data Analysis in the Behaviorial Sciences* (Psychology Press, ed. 0, 2014; https://www.taylorfrancis.com/books/9781317759980).

15. H. J. Seltman, *Experimental Design and Analysis* (Illinois Institute of Technology, 2018; http://hdl.handle.net/10560/islandora:1012018).

16. D. Broockman, J. Kalla, C. Caballero, M. Easton, "Political practitioners poorly predict which messages persuade the public" (preprint, Open Science Framework, 2023); https://doi.org/10.31219/osf.io/8un6a.

17. L. Hewitt, D. Broockman, A. Coppock, B. Tappin, J. Slezak, V. Coffman, N. Lubin, M. Hamidian, How Experiments Help Campaigns Persuade Voters: Evidence from a Large Archive of Campaigns' Own Experiments. *American Political Science Review* (forthcoming).

18. K. L. Milkman, L. Gandhi, M. S. Patel, H. N. Graci, D. M. Gromet, H. Ho, J. S. Kay, T. W. Lee, J. Rothschild, J. E. Bogard, I. Brody, C. F. Chabris, E. Chang, G. B. Chapman, J. E. Dannals, N. J. Goldstein, A. Goren, H. Hershfield, A. Hirsch, J. Hmurovic, S. Horn, D. S. Karlan, A. S. Kristal, C. Lamberton, M. N. Meyer, A. H. Oakes, M. E. Schweitzer, M. Shermohammed, J. Talloen, C. Warren, A. Whillans, K. N. Yadav, J. J. Zlatev, R. Berman, C. N. Evans, R. Ladhania, J. Ludwig, N. Mazar, S. Mullainathan, C. K. Snider, J. Spiess, E. Tsukayama, L. Ungar, C. Van den Bulte, K. G. Volpp, A. L. Duckworth, A 680,000-person megastudy of nudges to encourage vaccination in pharmacies. *Proceedings of the National Academy of Sciences* **119**, e2115126119 (2022).
